# Supplementary material for: Collaborative intelligence in AI: Evaluating the performance of a council of AIs on the USMLE
Source: PLOS Digit Health. 2025 Oct 9;4(10):e0000787. doi: 10.1371/journal.pdig.0000787 (PMC12510544; doi:10.1371/journal.pdig.0000787)
Supplement: S2 File — (ZIP) [file pdig.0000787.s002.zip › S2 Data Raw data files containing unprocessed question inputs and LLM outputs including transcripts of deliberations/STEP3_Question-99-1_Type-MC-NJ.docx]

**Facilitator** **[Round 1]:** A 36-year-old woman is referred to the office for evaluation of a fasting serum total cholesterol concentration of 249 mg/dL. She has a family history of early coronary artery disease (CAD) and her father died suddenly at age 46 years of myocardial infarction. She tells you that she has never had chest pain. She is not currently sexually active and has no children. The patient works as an advertising executive. She claims that her high-stress lifestyle makes it impossible for her to eat regular meals or to follow a special diet, and she usually eats fast food. She exercises two or three times a week for about 20 minutes on a treadmill. She has smoked one pack of cigarettes daily for the past 20 years. Her only medication is acetaminophen for tension headaches. She is 165 cm (5 ft 5 in) tall and weighs 76 kg (167 lb); BMI is 28 kg/m². Vital signs today are normal. Physical examination discloses no abnormalities except for mild obesity. Institution of which of the following is the most essential step in the prevention of CAD in this patient? (A) Biofeedback-based stress reduction program (B) More rigorous and consistent exercise program (C) Smoking cessation program (D) Strict low-calorie diet (E) Strict low-fat diet

**AI_A:** The question aims to determine the most pertinent step to take in preventing Coronary Artery Disease (CAD) in this specific patient, a woman with elevated cholesterol levels, family history of CAD, high stress levels, unhealthy eating habits, and a smoking habit. CAD is a condition where cholesterol-rich plaques obstruct the coronary arteries causing myocardial ischemia, which can lead to angina and myocardial infarction. Elevated cholesterol, stress, poor diet, lack of physical activity, and smoking are all recognized risk factors for CAD. Our assumptions could be that each of the provided options might contribute positively to the patient's health. Biofeedback-based stress reduction could aid in managing her stress levels, more frequent exercise could contribute to general heart health and maintenance of a healthy BMI, smoking cessation could lower the risk of CAD, and both diet options could help reduce cholesterol levels. However, we need an alternative assumption, given that we must prioritize one of these options. We know that smoking has been identified as a major risk factor for CAD, contributing to endothelial damage and promoting atherogenesis, and its cessation greatly reduces the risk for CAD. Thus, even in the presence of other risk factors, smoking cessation might be a priority. Clinically, of all mentioned risk factors, smoking is strongly associated with CAD. This association is due to its role in promoting the harmful modification of LDL cholesterol, causing oxidative stress and inflammation, promoting clotting factors, and reducing HDL cholesterol, which is protective. In conclusion, even though all provided options contribute to reducing the risk of CAD, some options contribute more significantly. Given the impact of smoking on the development of CAD and considering the patient's 20-year smoking history, it would be reasonable to prioritize the cessation of smoking as the most essential step in preventing CAD in this patient. Therefore, the answer is (C) Smoking cessation program.

**AI_E:** RESTATING THE QUESTION: The query seeks to know the most essential step to be taken in the preventive efforts for coronary artery disease (CAD) for a 36-year-old woman with a family history of CAD, high cholesterol, an inactive lifestyle, a high-stress job that disrupts her eating habits, mild obesity, and a daily smoking habit. Coronary Artery Disease, or CAD, is a condition where the major blood vessels supplying the heart get damaged or diseased. PLANNING: In deciding the best preventive step for the patient, we need to identify potential risk factors for CAD. History indicates a familial tendency, unhealthy eating habits, smoking, limited physical exercise, and being mildly obese. All these factors contribute to the development of CAD but not all are equally significant. A smoking cessation program, a more extensive and consistent exercise routine, restrictive calorie or fat diets, and a stress management program all seem like potential options. However, we need to prioritize which factor would result in the highest reduction in CAD risk. CLINICAL REASONING: Risk reduction in CAD often involves recognizing and managing modifiable risks. In this case, family history and age are non-modifiable. However, the patient's smoking habit, diet, lack of regular exercise, and stress are changeable elements. Amongst these, smoking has been proven to impart the highest risk for cardiovascular diseases out of all individual lifestyle factors. Smoking cessation dramatically reduces the risk of developing CAD over time. Although a well-rounded lifestyle alteration including diet, exercise, and stress management is ideal, the foremost priority in this patient's case would be smoking cessation. CONCLUDING: Given this patient's existing risk factors, the institution of a smoking cessation program, despite other worthy considerations like diet and exercise changes, would initially contribute the most to preventing CAD, considering the high attributable risk of tobacco use in cardiovascular disease. RESPONSE: Consequently, the correct answer would be (C) Smoking cessation program. This program would provide the most substantial immediate reduction in CAD risk, especially considering the patient's history.

**AI_D:** RESTATING THE QUESTION: The query is attempting to determine the best course of action for preventing coronary artery disease (CAD) in a 36-year-old woman who has a heightened risk due to an elevated total cholesterol level, a family history of early CAD, and a number of lifestyle risk factors which include obesity, smoking, high-stress, irregular unhealthy diet and inadequate exercise. The options presented range from stress reduction, increased exercise, smoking cessation and dietary adjustments. PLANNING: The major assumptions underlying the question appear to be that the woman's risk factors all contribute to her likelihood of CAD and that modifying any one of them could potentially reduce her risk. However, it's crucial to consider which of these risk factors have the greatest impact on CAD onset. A focused look at epidemiological and pathophysiological data shows that smoking tends to be one of the most significant risk factors for CAD. CLINICAL REASONING: Let's dive more deeply into these risk factors' relative contributions to CAD. To start, her total cholesterol level of 249 mg/dL is indeed high and could contribute to CAD. She also has a genetic predisposition to early CAD, which is also a substantial risk. Her diet, exercise routine, and high-stress lifestyle can aggravate this by contributing to obesity and increased cholesterol. However, among all these risks, the negative effects of smoking on cardiovascular health, particularly in terms of inducing atherosclerosis and increasing the risk of thrombosis, is very significant. Smoking can lead to endothelial injuries in arterial walls, favor lipid accumulation, and create a pro-thrombotic state. These factors hasten atherosclerotic plaque formation leading to CAD. CONCLUDING: Evaluating each of her risk factors then indicates that her smoking habit is a critical contributor to her overall risk. While addressing all of these risk factors could benefit her health more comprehensively, it seems that addressing her smoking habit should be the priority when it comes to preventing CAD specifically. RESPONSE: Therefore the most essential step in the prevention of CAD in this patient is cessation of smoking (C). It's important for her to understand that while making healthy lifestyle adjustments will be beneficial in managing her cholesterol and reducing her overall cardiovascular risk, ceasing smoking should be the primary clinical priority.

**AI_C:** RESTATING THE QUESTION: We have a 36-year-old woman who presents with total cholesterol levels above the acceptable range and has a significant history putting her at risk for coronary artery disease (CAD) including a family history of CAD and lifestyle factors like smoking, fast food diet, and high-stress job. Coronary artery disease is a type of cardiovascular disease where there's a buildup of plaque in the coronary arteries which can ultimately lead to a heart attack. The question is asking about the most essential step we can take to prevent CAD in this patient from the given options. PLANNING: Each of the given options contributes to general health and could potentially be beneficial to this patient. However, not all risk mitigating activities carry the same weight when considering CAD specifically. Risk factors for developing CAD include high levels of cholesterol, high blood pressure, smoking, diabetes, obesity, physical inactivity, and family history of early onset heart disease. Of these, certain risk factors like smoking, cholesterol levels, and physical inactivity are of higher priority in the context of CAD prevention. CLINICAL REASONING: Given that cholesterol levels in our patient are already high, measures related to dietary modification no doubt carry some weight. However, another more prominent risk factor is her smoking habit. Studies have shown that smoking significantly increases the risk of CAD due to its impact on heart rate, blood pressure, and clot formation. At the same time, evidence suggests that smoking cessation can greatly reduce the risk of CAD even in those who have been long-time smokers. CONCLUDING: Even though things like diet modification and stress reduction could indeed benefit this patient overall, when considering specifically the prevention of CAD, smoking cessation would be expected to have a more profound impact. Smoking is a leading cause of CAD and a risk factor that can be immediately acted upon. Thus, from among the given options, instituting a smoking cessation program would likely bring the most significant improvement in this patient's CAD risk profile. RESPONSE: Therefore, the key step in preventing CAD in this woman, as per the given options, would be (C) Smoking cessation program.

**AI_B:** RESTATING THE QUESTION: The question wants to know the most critical intervention that can help a 36-year-old woman prevent the onset of coronary artery disease (CAD). The woman, who has a family history of early CAD and high total cholesterol levels, leads a high-stress lifestyle, eats primarily fast food, exercises sporadically, and smokes creatively. Her only discernible medical issue, aside from high cholesterol, is mild obesity. PLANNING: We have several assumptions to consider. (1) High-stress lifestyle and poor nutrition may contribute to high cholesterol and obesity; however, many individuals with these lifestyles do not develop CAD. (2) Exercise aids in maintaining a healthy weight and may assist in managing cholesterol levels, yet it's not always sufficient to prevent CAD, particularly in the context of a high-risk lifestyle and genetic predisposition. (3) Smoking is a significant risk factor for CAD due to its effects on blood vessels and blood pressure. Smoking cessation could dramatically decrease her CAD risk. (4) While a low-fat or low-calorie diet may promote weight loss and manage cholesterol, adherence could be a challenge given her lifestyle, and significant dietary changes alone might not sufficiently lower her risk. From a clinical perspective, smoking cessation should be prioritized due to its direct link to CAD and its potential to greatly decrease her overall CAD risk when combined with other lifestyle changes. CLINICAL REASONING: As a result of nicotine, smoking causes vasoconstriction and increased heart rate, raising blood pressure and the heart's oxygen demand. Additionally, it contributes to atherosclerotic plaque formation, another risk factor for CAD. Given her daily smoking habit, discontinuation would likely result in significant cardiovascular benefits. Despite her poor diet and sporadic exercise, smoking is directly linked to CAD's pathophysiology and is a modifiable risk factor. While she will still need assistance in managing her cholesterol and weight, smoking cessation is an immediate priority that could have a significant impact on her cardiovascular health. CONCLUDING: In considering the risk factors involved in this patient's case - including cholesterol levels, family history, lifestyle, and smoking habits – the most significant and controllable factor for preventing CAD would be cessation of smoking. Hence, I would suggest the institution of a smoking cessation program as the critical step in preventing CAD for this patient. RESPONSE: (C) Smoking cessation program.

**Facilitator** The question seeks to identify the most crucial step in preventing Coronary Artery Disease (CAD) in a 36-year-old woman with several risk factors, including elevated cholesterol levels, a family history of CAD, high stress levels, unhealthy eating habits, sporadic exercise, and a smoking habit. All the given options could potentially contribute to improving the patient's health. However, the consensus among the AI responses is that smoking cessation would be the most impactful intervention for preventing CAD in this patient. Smoking is a significant risk factor for CAD due to its effects on blood vessels, blood pressure, and clot formation. It promotes the harmful modification of LDL cholesterol, causes oxidative stress and inflammation, promotes clotting factors, and reduces HDL cholesterol, which is protective. Therefore, cessation of smoking would likely result in significant cardiovascular benefits and greatly reduce her CAD risk. While other lifestyle changes such as diet modification, stress reduction, and increased exercise are important for overall health and could assist in managing her cholesterol levels and weight, they may not have as immediate or significant an impact on her cardiovascular health as smoking cessation. Therefore, the most essential step in the prevention of CAD in this patient, given the options, would be the institution of a smoking cessation program (C).
